# Comparison of photoinduced and electrochemically induced degradation of venlafaxine

**DOI:** 10.1007/s11356-024-32018-5

**Published:** 2024-01-22

**Authors:** Melanie Voigt, Jean-Michel Dluziak, Nils Wellen, Victoria Langerbein, Martin Jaeger

**Affiliations:** Department of Chemistry and ILOC, Niederrhein University of Applied Sciences, Frankenring 20, D-47798 Krefeld, Germany

**Keywords:** Advanced oxidation processes, LC-HRMS, Venlafaxine, Electrochemical oxidation, Photoinduced degradation, QSAR

## Abstract

The European Union requires environmental monitoring of the antidepressant drug venlafaxine. Advanced oxidation processes provide a remedy against the spread of micropollutants. In this study, the photoinduced and electrochemical decompositions of venlafaxine were investigated in terms of mechanism and efficacy using high-performance liquid chromatography coupled to high-resolution multifragmentation mass spectrometry. Kinetic analysis, structure elucidation, matrix variation, and radical scavenging indicated the dominance of a hydroxyl-mediated indirect mechanism during photodegradation and hydroxyl and direct electrochemical oxidation for electrochemical degradation. Oxidants, sulfate, and chloride ions acted as accelerants, which reduced venlafaxine half-lives from 62 to 25 min. Humic acid decelerated degradation during ultra-violet irradiation up to 50%, but accelerated during electrochemical oxidation up to 56%. *In silico* quantitative structure activity relationship analysis predicted decreased environmental hazard after advanced oxidation process treatment. In general, photoirradiation proved more efficient due to faster decomposition and slightly less toxic transformation products. Yet, matrix effects would have to be carefully evaluated when potential applications as a fourth purification stage were to be considered.

**Supplementary Information:**

The online version contains supplementary material available at 10.1007/s11356-024-32018-5.

## Introduction

The antidepressant venlafaxine has been detected in the aquatic environment worldwide. It has hence been included by the Commission of the European Union in the 3rd and 4th EU Watch List to assess the Europe-wide distribution and hazard to the aquatic environment (European Commission 2020, 2022). The database of the Federal Environment Agency Germany reports over 400 findings of venlafaxine in various water bodies (Dusi et al. 2019). Concentrations varied between 0.02 ng/L and 400 μg/L. The highest concentrations, i.e., above 1 μg/L, were observed in surface water and wastewater treatment plants (Dusi et al. 2019). The main routes of entry into the aquatic environment are conventional wastewater treatment plants, which are not capable of completely eliminating venlafaxine from sewage water. Venlafaxine acts as an antidepressant, which is partially metabolized when ingested and is excreted in the urine and thus enters the wastewater. Improper disposal in the toilet also leads to venlafaxine entering the water. Several studies were dedicated to the ecotoxicity assessment of venlafaxine (Galus et al. 2013; Kumar et al. 2022). It was found harmful to, e.g., zebrafish *Danio rerio*, where the embryo production decreased significantly after a 6-week exposure to a pharmaceutical convolute including venlafaxine.

In order to reduce the amounts of pollutants and thus eliminate the danger to the aquatic environment, various methods have been researched. So-called advanced oxidation processes (AOPs) have been considered very promising to achieve complete elimination of the unwanted substances (Cuerda-Correa et al. 2020; Wang and Zhuan 2020; Coha et al. 2021). These AOPs include ozonation, UV irradiation, sonolysis, and electrochemical processes, referred to as EAOPs, where degradation is mediated through electrochemical oxidation (Deng and Zhao 2015; Voigt et al. 2020). Common to AOPs and EAOPs is the occurrence of hydroxyl radicals which possess a strong oxidation potential of 2.8 V and can hence induce the decomposition of substances such as pharmaceuticals and pesticides (Parsons 2004). Ideally, a complete mineralization of the substance takes place (Albornoz et al. 2021; Cano et al. 2020; Poulopoulos et al. 2021). In some cases, transformation or degradation products may be produced that turn out more toxic than the initial substances (Fatta-Kassinos et al. 2011). While the hydroxyl radical mechanism is often the major mode of action of AOPs, directly induced oxidation, e.g., following photoexcitation or electron transfer, has been reported (Voigt and Jaeger 2021; Voigt et al. 2022a).

**Fig. 1:**
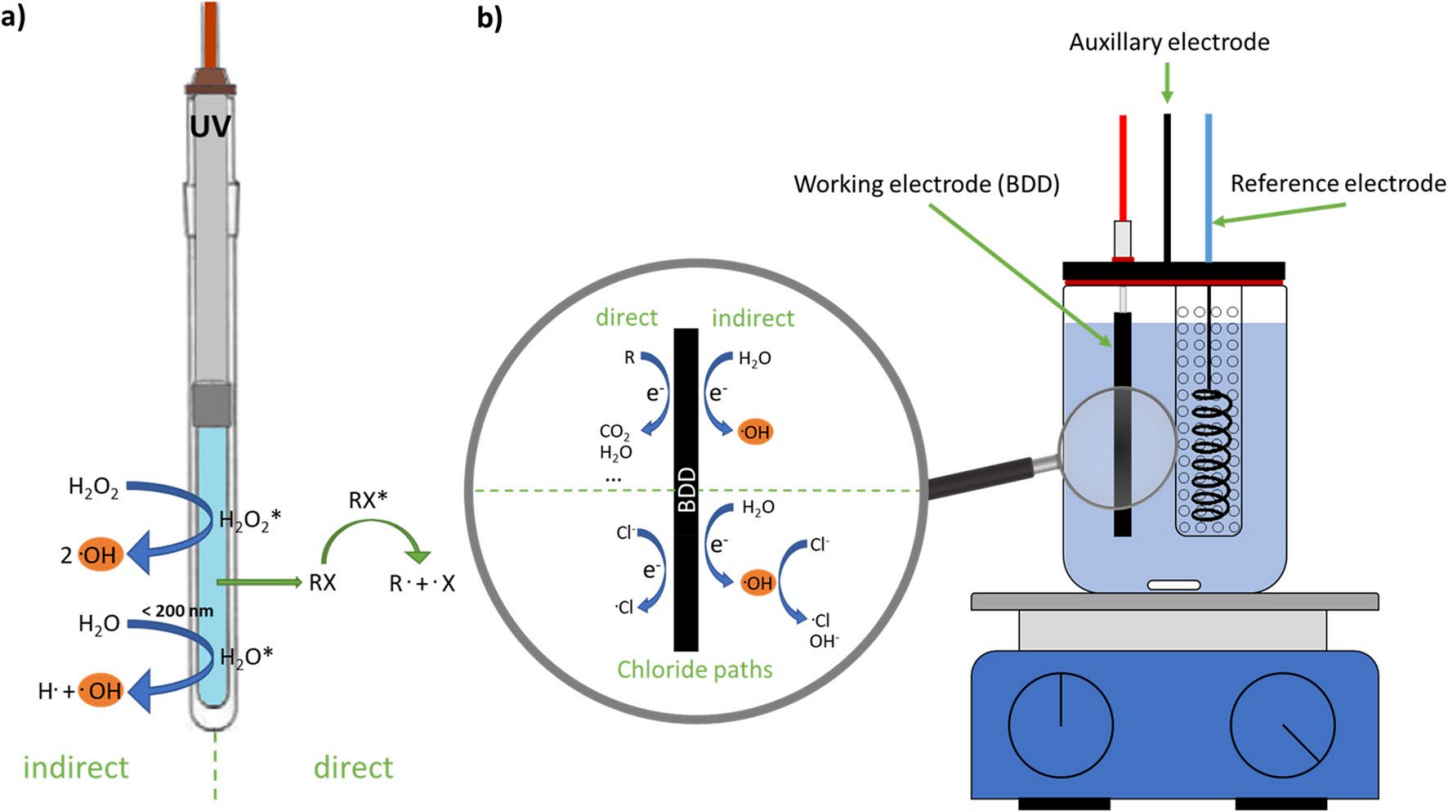
Scheme of UV lamp (**a**) and electrolytic cell (**b**) with illustrations of the corresponding direct and indirect mechanism of transformation processes. Two additional pathways are indicated for electrolysis in the presence of chloride ions

Figure 1a illustrates the two pathways for photoinduced degradation (Zhang et al. 2017; Lastre-Acosta et al. 2019; Voigt et al. 2022b). Hydrogen peroxide may be added, thus increasing hydroxyl radical concentration and enhancing the indirect mechanism. Upon the addition of a radical scavenger, such as *tert*-butanol and methanol, the indirect pathway is blocked, leaving only the direct mechanism at work. Products resulting from the direct mechanism would hence dominate (Voigt et al. 2022a, 2022b). Both pathways have been reported for EAOPs as well (Nidheesh Jum et al. 2018; Marinho et al. 2022). Hydroxyl radicals are formed from water molecules that adsorb on the electrode surface (see Fig. 1b) (Moreira et al. 2017; Nidheesh et al. 2018; Marinho et al. 2022). Analogously, the substances adsorb on the surface of the electrode and experience electron transfer and degradation is initiated. To enhance efficacy, ions such as sulfate, phosphate, carbonate, or chloride are added to the solution (Nidheesh et al. 2018; Marinho et al. 2022). Boron-doped diamond (BDD) electrodes are particularly suitable for the generation of hydroxyl radicals (Moradi et al. 2020). Similar to hydrogen peroxide reacting with UV radiation, the ions can directly be reduced to reactive species. Otherwise, they can react with hydroxyl radicals to form second-generation radicals and induce the destruction of the pollutants. A comparative compilation of the four EAOP-induced pathways is given with chloride as an example in Fig. 1b.

Most reported studies used laboratory conditions. To emulate real conditions, humic or fulvic acid considered suitably representing natural organic matter (NOM) in water matrices was applied (Slomberg et al. 2017).

AOPs may lead to increased ecotoxicity resulting from the transformation of products. Ecotoxicity can be assessed through *in vivo*, *in vitro*, and *in silico* methods. *In vivo* assays rely on living organisms, which are often killed. Furthermore, photoinduced degradation or electrochemical oxidation often generates products that are not commercially available as reference standards since their synthesis may prove complex and costly. This unavailability obstructs both *in vivo* and *in vitro* testing (Sakkiah et al. 2018; Tang et al. 2018; Satpathy 2019; Hou et al. 2020). Therefore, *in silico* QSAR analysis is becoming more and more popular. QSAR analyses create models from experimental data of similar compounds to predict properties, such as ecotoxicity (Sakkiah et al. 2018; Tang et al. 2018; Satpathy 2019; Schneider et al. 2019; Voigt and Jaeger 2023). For this purpose, structural similarities are identified on the basis of the simplified molecular input-line entry system (SMILES) of a molecule. Among already established models for ecotoxicity prediction using QSAR analysis is the Ecological Structure Activity Relationship (ECOSAR) (Mayo-Bean et al. 2017; Wright et al. 2022; Voigt and Jaeger 2023). Models were created based on the largest database of curated ecotoxicity data ECOTOXicology Knowledgebase (ECOTOX) maintained by the US Environmental Protection (EPA). The prediction is achieved based on the structural similarities (Olker et al. 2022; Voigt and Jaeger 2023).

In this study, UV radiation–induced degradation and electrochemical oxidation were compared for the pharmaceutical micropollutant venlafaxine. One focus was laid on the identification of the transformation products by high-performance liquid chromatography coupled to high-resolution multifragmentation mass spectrometry (HPLC-HRMS) to distinguish between the active mechanisms. To this purpose, the influence of additives and matrices on the mechanism was investigated using *tert*-butanol as a radical scavenger, hydrogen peroxide, sulfate, and chloride as oxidants. As the NOM model humic acid was chosen. For the electrochemical oxidation, a standard BDD electrode was used. Additionally, the influence of pH and two ions was studied. For comparative ecotoxicity assessment, QSAR analyses based on the identified transformation product structures were subsequently performed.

## Materials and methods

### Chemicals and reagents

Venlafaxine hydrochloride (>= 97.5%) and *tert*-butanol (99.5%) were purchased from Thermo Fisher Scientific (Geel, Belgium). Sulfuric acid (95–97%, pro analysis) and hydrochloric acid (37%, pro analysis) were received from Bernd Kraft GmbH (Duisburg, Germany). Ammonia (approximately 25%) was acquired from Honeywell Specialty Chemicals Seelze GmbH (Seelze, Germany). *Tert*-Butanol (Acros Organics, Geel, Belgium) was used as a radical scavenger. For natural organic matter simulation, humic acid was purchased from Alfa Aesar (≥98%, Haverhill, MA, USA). Hydrogen peroxide was used as a 30% stabilized H_2_O_2_ solution (Carl Roth, Karlsruhe, Germany). Acetonitrile was obtained from Carl Roth (Karlsruhe, Germany). Formic acid (FA) was received from Fluka-Honeywell (Seelze, Germany).

### Absorption spectra

Absorption spectra of 20 mg/L venlafaxine dissolved in ultra-pure water (Berrytec, Grünwald, Germany) were recorded using a UV5Nano spectrometer (Mettler Toledo, Columbus, USA). Emission spectra of the UV lamp were acquired using a HR4000 spectrometer (Ocean Optics, Duiven, The Netherlands).

### Photodegradation experiments

All photoinduced degradation experiments were carried out in a 1 L batch reactor (Peschl Ultraviolet; Mainz, Germany), which was equipped with a low-pressure mercury UVC lamp (TNN 15/32, 15 W, Heraeus, Hagen, Germany) at its center. The UVC lamp emitted at wavelengths of 185, 254, 313, 365, 405, 437, 547, 578, and 580 nm, with 254 nm as a major UV emission (25%). The photon fluence rate was determined by ferrioxalate actinometry to 2.03 mmol min^−1^ for the UVC lamp (Hatchard and Parker 1956; Kuhn et al. 2004; Bolton et al. 2011). Electron spin resonance spectroscopy using spin traps was applied to prove the occurrence of hydroxyl radicals (Kochany and Bolton 1991, 1992; Sun and Bolton 1996). The reactor was encased with aluminum foil.

Solutions of venlafaxine in ultra-pure water with a final concentration of 20 ± 0.7 mg L^−1^ were poured into the photoreactor. The pH values were between 5.4 and 7.0 at the beginning of the experiment and decreased to 3.9 and 4.1 during 10 min of UV irradiation. *Tert*-butanol was added to the solution for radical scavenging experiments to yield a final concentration of 10 vol-% and 30 vol-%. For studying the influence of dissolved organic matter, 5 mg L^−1^, 10 mg L^−1^, and 30 mg L^−1^ of humic acid (HA) were added to solutions of venlafaxine. Hydrogen peroxide was added to yield final concentrations of 10 mg L^−1^ and 30 mg L^−1^ which were checked using Merckoquant peroxide test strips (Merck, Darmstadt, Germany).

The temperature was constant at 22 ± 2 °C without additional cooling. The solution was stirred using a magnetic stirrer at about 500 rounds per minute. During the first 5 min of UV irradiation, samples were taken from the reactor every 30 s, then every minute for a further 5 min. The resulting 16 samples were analyzed using HPLC-HRMS. For this purpose, samples were taken from the photoreactor using a syringe and transferred into a 2 mL vial.

### Electrochemical oxidation experiments

Electrochemical oxidation experiments were carried out in a ROXY^TM^ system (Antec Scientific, Zoeterwoude, The Netherland), which consists of a small-scale electrosynthesis cell (SynthesisCell, Antec Scientific, Zoeterwoude, The Netherland) and a potentiostat. This system was controlled with Dialogue Elite^TM^ software (Antec Scientific, Zoeterwoude, The Netherlands) version 2.21.8.1.

A solution containing 20 ± 0.3 mg L^−1^ venlafaxine in ultra-pure water was prepared. For adjusting pH values, formic acid and ammonia were used. To investigate the influence of sulfate and chloride ions, sulfuric acid and hydrochloric acid were added until the solution reached pH 3. As radical scavengers, *tert*-butanol was added to final concentrations of 10% and 30%. For samples simulating NOM matrices, humic acid was used to yield 5 mg/L. Hydrogen peroxide was applied at final concentrations of 10 mg L^−1^ and 30 mg L^−1^. Conductivity was measured with a pHenomenal® CO 3100 pH conductometer (VWR International, Radnor, PA, USA).

Solutions were electrochemically oxidized using the BDD electrode. Samples of 1mL were taken after 0, 10, 20, 30 45, 60, 75, 90, 120, 150, 180, 210, 240, 300, and 360 min using a syringe. The voltage was kept at 1.5 V. The samples were transferred to HPLC-HRMS.

### HPLC-HRMS analysis

Sample analysis was investigated directly after sampling using an electrospray ionization-quadrupole-ion trap-orbitrap (Orbitrap IDX, Thermo Fisher Scientific, Waltham, USA) coupled to an ultra-high-performance liquid chromatography instrument (Vanquish Core, Thermo Fisher Scientific, Waltham, USA). The injection volume was 5 μL. Reversed-phase chromatographic analysis was performed using an Eclipse Plus C18 (ZORBAX, 3.5 μm, 2.1 × 150 mm, Agilent, Waldbronn, Germany). During 10 min, the chromatography was performed isocratically 80% eluent A and 20% eluent B at a flow rate of 0.3 mL min^−1^. Eluents were ultra-pure water as A and acetonitrile as B both acidified with 0.1% formic acid.

The mass range was selected from 100 to 2000 *m/z*. The resolution was set to 60,000. A collision energy of 30 eV and a resolution of 30000 were chosen for MS^2^. For MS^3^, 45 eV and a resolution of 60,000 were set. Fragmentation was performed in the higher-energy collision-induced dissociation (HCD) cell. The spray voltage was set to 3500 V, and the vaporizer and ion transfer tube temperature was 300 °C. Instruments were controlled using Thermo Scientific XCalibur Version 4.3.73.11.

For structure elucidation, accurate mass and isotope pattern were recorded allowing us to derive the molecular formula. Tentative structures were generated using the software ACD/ChemSketch 2016.1.1 (ACDLabs, Toronto, ON, Canada). Based on the initial chemical structure, plausible structures were identified. As the next step, MS/MS and MS^3^ spectra were inspected. Observed *m/z* values and differences were compared with expectancy values of fragments created from the plausible structures according to the standard fragmentation rules (Niessen 2011; Niessen and Honing 2015; Schymanski et al. 2015). Best matches with respect to fragmentation pathway and *m/z* values were considered structurally confirmed.

### QSAR

For an estimation of the ecotoxicity of the identified products, QSAR analyses were performed using the OECD QSAR toolbox Version 4.3.1 (OECD, Paris, France). The model Ecological Structure Activity Relationship (ECOSAR) was chosen. First, the chemical structure formulae of venlafaxine and its transformation and degradation products were sketched using ACD/ChemSketch 2016.1.1 (ACDLabs, Toronto, ON, Canada) software and imported into the QSAR toolbox. Within ECOSAR, the chemical structures were employed in the SMILES representation. Depending on the SMILES combination, the chemical substances were assigned to different classes. Chronic toxicity as chronic value (ChV) and acute toxicity as lethal concentration, 50% (LC_50_), and half maximal effective concentration (EC_50_) were predicted. As organisms, *Daphnia* (Branchiopoda), fish (Actinopterygii), and green algae were chosen.

## Results and discussion

### Photoinduced degradation of venlafaxine

**Fig. 2:**
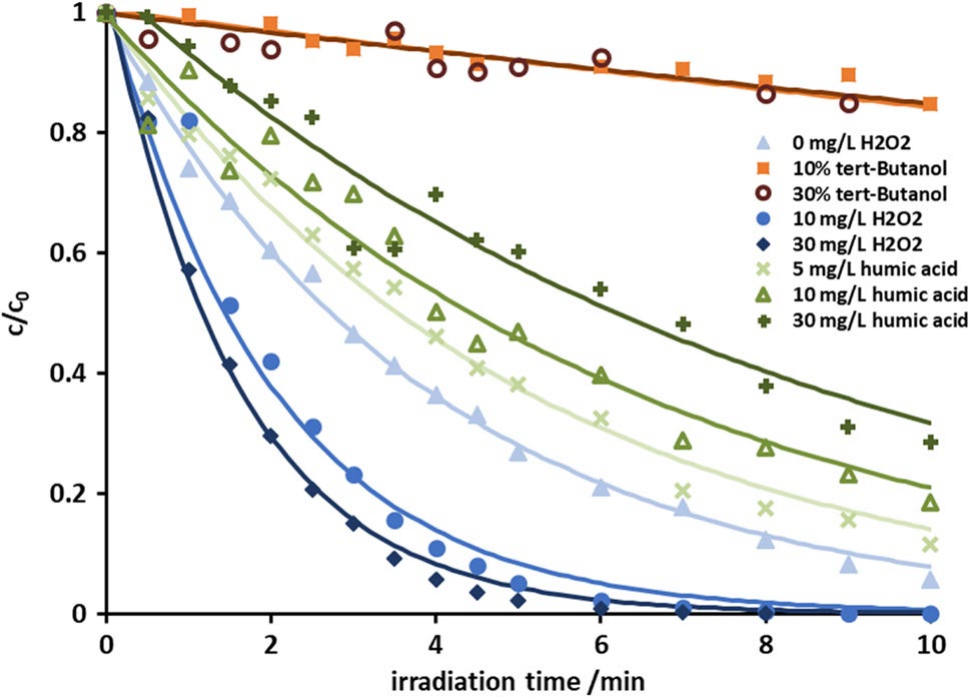
Normalized concentration-time curves of venlafaxine in pure water (light blue filled triangle), in the presence of H_2_O_2_ 10 mg/L (blue filled circle) and 30 mg/L (dark blue filled diamond), of humic acid 5 mg/L (×, light green), 10 mg/L (green open triangle), with 30 mg/L (+, dark green), of *tert*-butanol 10% (orange filled square) and 30% (brown open circle); initial concentration of venlafaxine was 20 ± 0.3 mg L^−1^. Error bars are given in the corresponding graphs in the SI

The absorption spectrum of venlafaxine and the emission spectrum of the UV lamp are given in the supplemental information (cf. Fig. S1). The emission band at 254 nm coincides with an absorption band of venlafaxine, indicating the potential for photoexcitation and subsequent direct degradation. Normalized concentration-time curves, i.e., mass area vs. irradiation time, of photoinduced degradation of venlafaxine are presented in Fig. 2 and Fig. S2. The thus-derived rate constants and the corresponding half-lives are collected in Table S1 in the supplemental information. The presence of hydrogen peroxide accelerated the decomposition, whereas the radical scavenger *tert*-butanol exercised strong deceleration.

While the addition of hydrogen peroxide augments the occurrence of hydroxyl radicals, a too-large excess of hydrogen peroxide, as was the case with 30 mg/L, did not significantly increase the degradation velocity. This was explained in terms of concentration increase would lead to higher probabilities for self-decomposition of hydrogen peroxide and recombination of hydroxyl radicals (Balakrishnan et al. 2020). This is referred to as supersaturation (Sun and Bolton 1996; Parsons 2004; Voigt and Jaeger 2017). The comparison between the degradation in ultra-pure water and the degradation after the addition of 5 mg humic acid is interesting. The majority of degradation experiments reported in various studies was carried out under laboratory conditions in ultra-pure water. The addition of 5 mg humic acid was found to represent the water matrix of surface water (data shown in supplemental information). Humic acid reduced the degradation velocity. This might be due to the radical scavenging properties or light absorption of humic acid thus reducing in both cases the amount of hydroxyl radicals. Further to diminishing the amount of radiation available for venlafaxine decomposition induction, humic acid itself was found to degrade upon irradiation (Tang et al. 2020). It can therefore be assumed that venlafaxine may degrade more slowly in natural or effluent waters when applying AOPs. Supersaturation also occurred upon the addition of *tert*-butanol. Yet, *tert*-butanol itself decomposes upon UV irradiation. Hence, hydroxyl radicals were less scavenged, and degradation of venlafaxine, albeit to a lesser extent, was observed. Taking the absorption spectrum into account, the degradation could also be due to a contribution of the direct mechanism. As the addition of substances with radical scavenging properties reduced the degradation velocity, the indirect mechanism was nevertheless assumed the dominant mechanism. At this stage, a contribution from the direct mechanism cannot be excluded. To address this issue, identification, structure elucidation, and quantitative estimation of the transformation products were employed, since products from the two pathways should be different and may be specific for the two pathways.

### Photoinduced transformation products of venlafaxine

The photoinduced transformation products were investigated using higher-order mass spectrometry after chromatographic separation. The observed and identified transformation products are collected in Table 1.

**Table 1:**
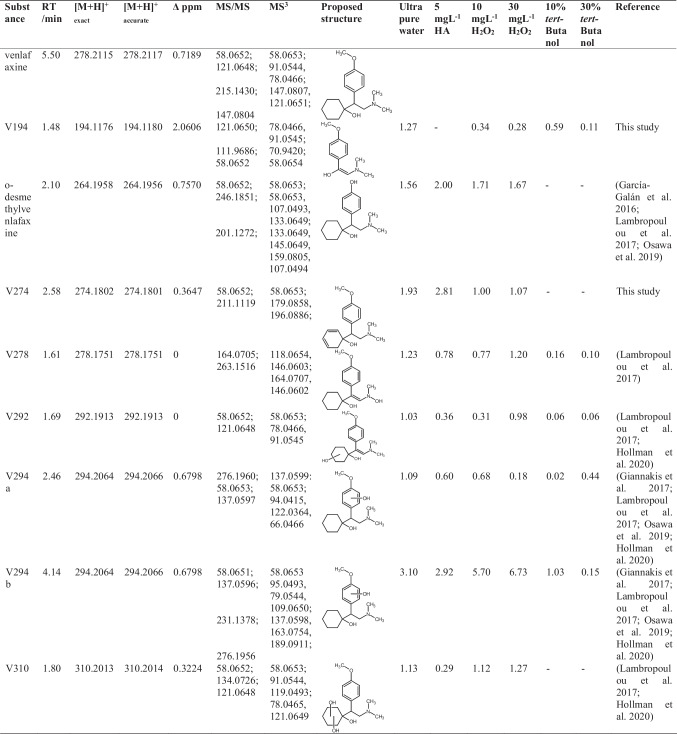
Observed and identified photoinduced degradation and transformation products of venlafaxine. Retention times (RT), quasi-molecular ion masses, mass error Δ ppm, major MS/MS, MS^3^ fragments, structure proposals, and matrix components (humic acid, hydrogen peroxide, *tert*-butanol) are given

A total of eight products were identified in ultra-pure water under UV irradiation, two of which have not yet been described in previous studies. Upon the addition of 30% *tert*-butanol, no transformation products were observed above a 1% threshold with respect to the initial substance. This fact was taken as indicative that products were formed via the indirect path, and thus the dominance of the hydroxyl radical–induced mechanism was evident. Inspecting the proposed structures, it can be recognized that hydroxyl groups were accumulated in the molecule, which is consistent with the indirect mechanism. No products point to the direct absorption pathway.

In the MS/MS spectra, all except product V278 showed the fragment with *m/z* = 58.0652 ± 0.001. This was interpreted as the dimethylamine fragment of venlafaxine, which remained stable. For V278, a methyl group was substituted by a hydroxyl group prohibiting the observation of the dimethylamine fragment. Another frequently observed fragment was characterized by *m/z* = 121.0649 ± 0.001. This was assigned to 1-methoxy-4-methylbenzene.

Giannakis et al. (2017) proposed different structures for observed ions in their study. These corresponded to isomers of V194, V292, and V310. García-Galán et al. (2016) and Osawa et al. (2019) observed o-desmethylvenlafaxine and further isomers of V274, V278, V292, V294, and V310. In all studies, MS/MS spectra were recorded, such that structure interpretation supports the occurrence of different isomers.

The products V294a and V294b had the same *m/z* ratios and appeared at different retention times. This observation suggested isomers, which can be traced back to different substitution positions of hydroxyl groups leading to different polarities of the products. For more detailed identification, MS^n^ spectra were applied. Yet, identical fragments were observed. It was hence assumed that the hydroxyl group was located at the aromatic ring. The exact position however could not be determined for either isomer.

**Fig. 3:**
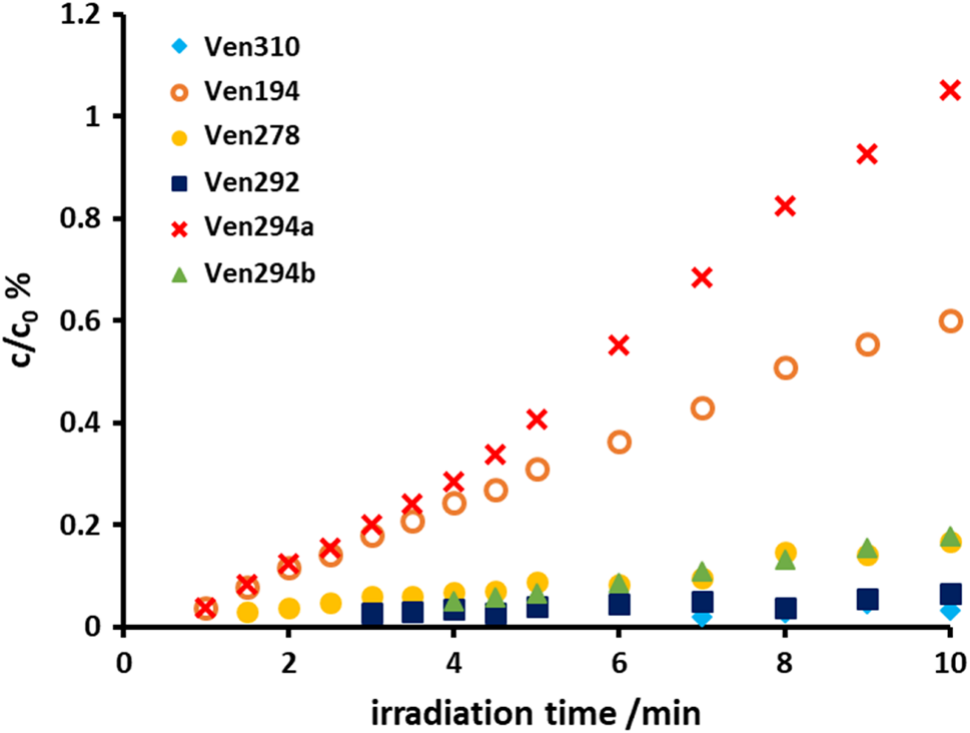
Normalized concentration-time diagram of venlafaxine V194 (orange open circle), V278 (yellow filled circle), V292 (dark blue filled square), V294a (×, red), V294b (green filled triangle), and V310 (light blue filled diamond) upon photoirradiation in the presence of 10% *tert*-butanol; initial concentration of venlafaxine was 20 ± 0.3 mg L^−1^. Error bars are given in the corresponding graphs in the SI

The compounds of Fig. 3 exhibited a prolonged formation in ultra-pure water in the presence of *tert*-butanol. They reached a maximum of 0.5% of the initial compound concentration after 10 min of UV irradiation. Figure 3 and S2 show the corresponding normalized concentration-time graphs in the presence of 10% *tert*-butanol.

The product formation proceeded approximately linearly in analogy to the degradation of venlafaxine (cf. Fig. 2). The course was interpreted as *tert*-butanol decomposing and hydroxyl radical concentration increasing after 10 min of irradiation. Hence, the concentration of the indirectly formed products increased slowly.

As a preliminary conclusion, photoinduced degradation of venlafaxine occurred exclusively via the indirect mechanism. There were no indications for the direct degradation mechanism. Thus, the formation of hydroxyl radicals in water will have a great influence on degradation and product formation. The application of EAOP and the mechanistic comparison will be discussed in the following.

### Electrochemical oxidation

**Fig. 4:**
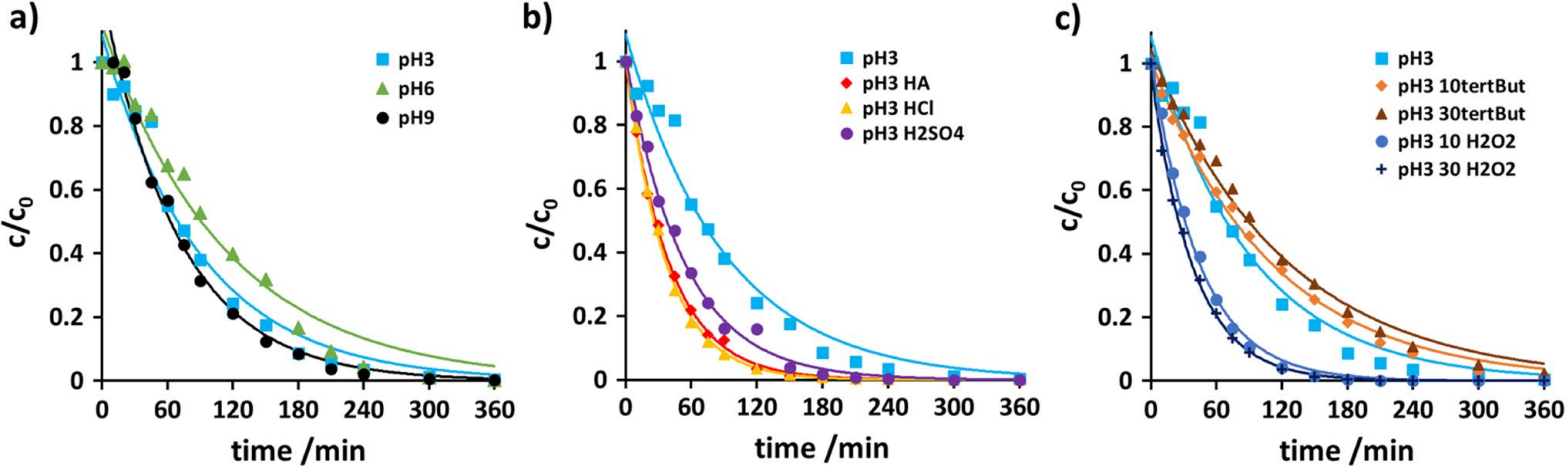
Normalized concentration-time curves of electrochemical oxidation of venlafaxine at **a** pH value 3 (light blue filled square), pH value 6 (green filled triangle), and pH value 9 (black filled circle), **b** in the presence of sulfuric acid (violet filled circle), hydrochloric acid (yellow filled triangle), and humic acid (red filled diamond) at pH 3; **c** in the presence of *tert*-butanol 10% (orange filled diamond) and 30% (brown filled triangle), and of hydrogen peroxide 10 mg/L (blue filled circle) and 30 mg/L (+, dark blue); initial concentration of venlafaxine was 20 ± 0.3 mg L^−1^. Error bars are given in the corresponding graphs in the SI

The electrochemical oxidation of venlafaxine under various conditions was monitored using HPLC-HRMS. The resulting exposure time-dependent mass areas were normalized and plotted as normalized concentration-time curves. They are displayed in Fig. 4.

All concentration-time curves obtained were best described according to first-order kinetics in agreement with previous studies (Jum et al. 2018). Compared to the photoinduced degradation experiments, total degradation was achieved after about 6 h, while photoinduced degradation was nearly completed after 10 min. The degradation was accelerated on the addition of sulfate and chloride ions as well as hydrogen peroxide. The addition of humic acid also showed an accelerating effect. A quantitative comparison was achieved via the rate constants and their corresponding half-lives as given in Table 2.

**Table 2:** Conductivity of the venlafaxine matrix solution, rate constants *k*, and corresponding half-lives *t*_0.5_ of venlafaxine for electrochemical oxidation

pH value	pH adjusting adjusted by:	*tert-*butanol/%	H_2_O_2_/mg L^−1^	*k*/min^−1^	*t*_0.5_/min	Conductivity/μS cm^−1^ @20 °C
3	Formic acid	0	0	1.12E−02	62.00	218
6	-	0	0	8.84E−03	78.39	7.1
9	Ammonia	0	0	1.43E−02	48.44	55.6
3	Sulfuric acid	0	0	1.88E−02	36.89	752
3	Hydrochloric acid	0	0	2.75E−02	25.18	303
3	Humic acid and formic acid	0	0	2.52E−02	27.55	370
3	Formic acid	10	0	9.11E−03	76.09	170
3	Formic acid	30	0	8.21E−03	84.43	194
3	Formic acid	0	10	2.30E−02	30.20	226
3	Formic acid	0	30	2.61E−02	26.56	234

Stability tests were performed to exclude degradation over time without electrochemical oxidation. Venlafaxine is stable at all three pH values.

The rate constants at different pH values showed that degradation was slowest at pH 6. This was due to the low conductivity. Degradation was faster at pH 3 and fastest at pH 9, despite the conductivity of the solution. Ammonia accelerated degradation, which was explained in previous studies as due to oxidation at the BDD electrode generating amino radicals (Kumari and Kumar 2023). In the presence of oxygen, the amino radicals give rise to amino peroxyl radicals, which react further to form different nitroxides as oxidants.

Ions, such as sulfate and chloride, led to acceleration, too, where chloride proved the better oxidant, compensating for the lower conductivity. Since structure elucidation did not reveal any chlorine or nitro-substituted transformation products, the indirect chloride mechanism, i.e., hydroxyl radical–mediated, appeared the predominant one. The analogue should apply for solutions containing ammonia.

Yet structure elucidation (cf. Table 3) did not reveal any chlorine or amino substituted transformation products, hence either the indirect chloride mechanism, i.e., hydroxyl radical–mediated, or the direct electrochemical oxidation would be predominant. The fastest venlafaxine disintegration was observed in the presence of chloride, 30 mg/L hydrogen peroxide, and 5 mg/L humic acid. Analogously to the photoinduced degradation, the electrochemical oxidation process was slowed down by adding *tert*-butanol, again suggesting that hydroxyl radicals may play a major role as oxidants. While supersaturation was observed again, the addition of hydrogen peroxide exercised a much stronger effect than was the case during irradiation.

**Table 3:**
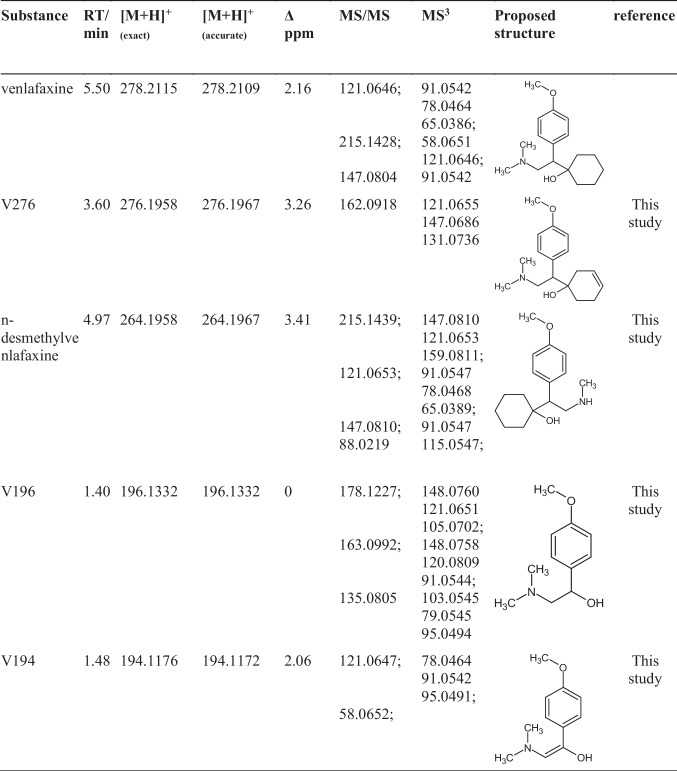
Observed and identified electrooxidation degradation and transformation products of venlafaxine. Retention times (RT), quasi-molecular ion mass-charge ratios, mass accuracy, MS/MS, MS^3^ fragments, and proposed structures

In general, it may be concluded that the conductivity of a solution accounted only for a small contribution to the efficiency of the electrochemical oxidation. The application of oxidants or hydrogen peroxide promoted the acceleration of the degradation. With respect to utilization as an advanced purification stage in wastewater treatment plants, it is interesting that the presence of humic acid rendered degradation faster in contrast to prolonging photoinduced degradation.

Structural inspection of the transformation products will shed light on the mechanistic details. The identified products are shown in Table 3.

A total of four transformation products with *m/z* = 276.1953, *m/z* = 264.1956, *m/z* = 196.1334, and *m/z* = 194.1177 were identified. None of them has previously been reported with electrochemical oxidation using a BDD electrode. The two products V276 and n-desmethylvenlafaxine were observed exclusively at pH 9. V194 was detected in all electrochemical oxidation experiments except at pH 9 containing ammonia. V196 and V194 were most likely formed through hydroxyl radicals, since the cyclohexanol moiety was substituted by a hydroxyl group. In contrast, V276 and n-desmethylvenlafaxine underwent reduction and demethylation, which may be traced back to the direct electrochemical oxidation mechanism. It is interesting to note that o-desmethylvenlafaxine was formed during photoinduced degradation but n-desmethylvenlafaxine during electrochemical oxidation. Both were observed at the same mass-charge ratio, but had different retention times. Distinction succeeded via MS^n^ fragmentation. As opposed to the photoinduced degradation of venlafaxine, indices for both the direct electrochemical oxidation and the hydroxyl radical–mediated indirect mechanism were found.

**Fig. 5:**
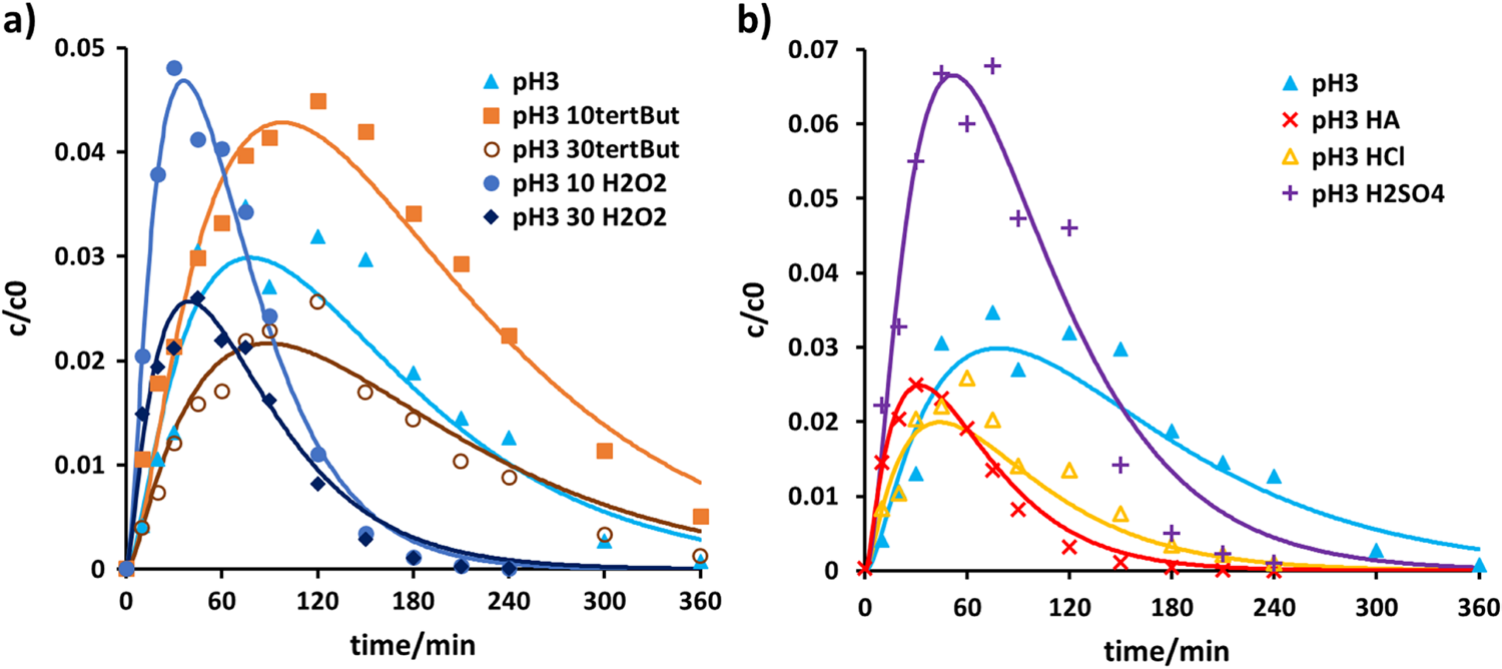
Concentration-time curves of V194 normalized to venlafaxine at pH 3 (light blue filled triangle); **a** in the presence of *tert*-butanol 10% (orange filled square), 30% (brown open circle), hydrogen peroxide 10 mg/L (blue filled circle), 30 mg/L (dark blue filled diamond) and **b** in the presence of sulfate (+, violet), chloride (yellow open triangle), and humic acid (×, red); initial concentration of venlafaxine was 20 ± 0.3 mg L^−1^. Error bars are given in the corresponding graphs in the SI

All observed products emerged in low concentrations during electrochemical oxidation. Yet, concentration-time profiles in relation to venlafaxine could be derived for the product V194 under various matrix conditions. The data could be best described following a subsequent follow-up reaction according to Eq. 4 in the supplemental information (see Fig. 5).

On hydrogen peroxide addition, V194 was formed and degraded faster, presumably due to increased hydroxyl radical concentrations. Deceleration was observed on the addition of radical scavengers, where the transformation product was formed after an extended period of electrochemical oxidation. Degradation despite the presence of *tert*-butanol may be explained in terms of *tert*-butanol degradation. A higher concentration of hydrogen peroxide or *tert*-butanol resulted in a lower amount of the product. The presence of chloride, sulfate, and humic acid was found to have an accelerating effect on both the formation and degradation of V194. Again, chloride ions resulted in the fastest decay of the transformation product V194 as was found for venlafaxine. Compared to photoinduced degradation, significantly fewer products were observed. In both processes, a maximum of 8% product formation was observed.

### QSAR analysis of photoinduced and electrochemical degradation products

**Fig. 6:**
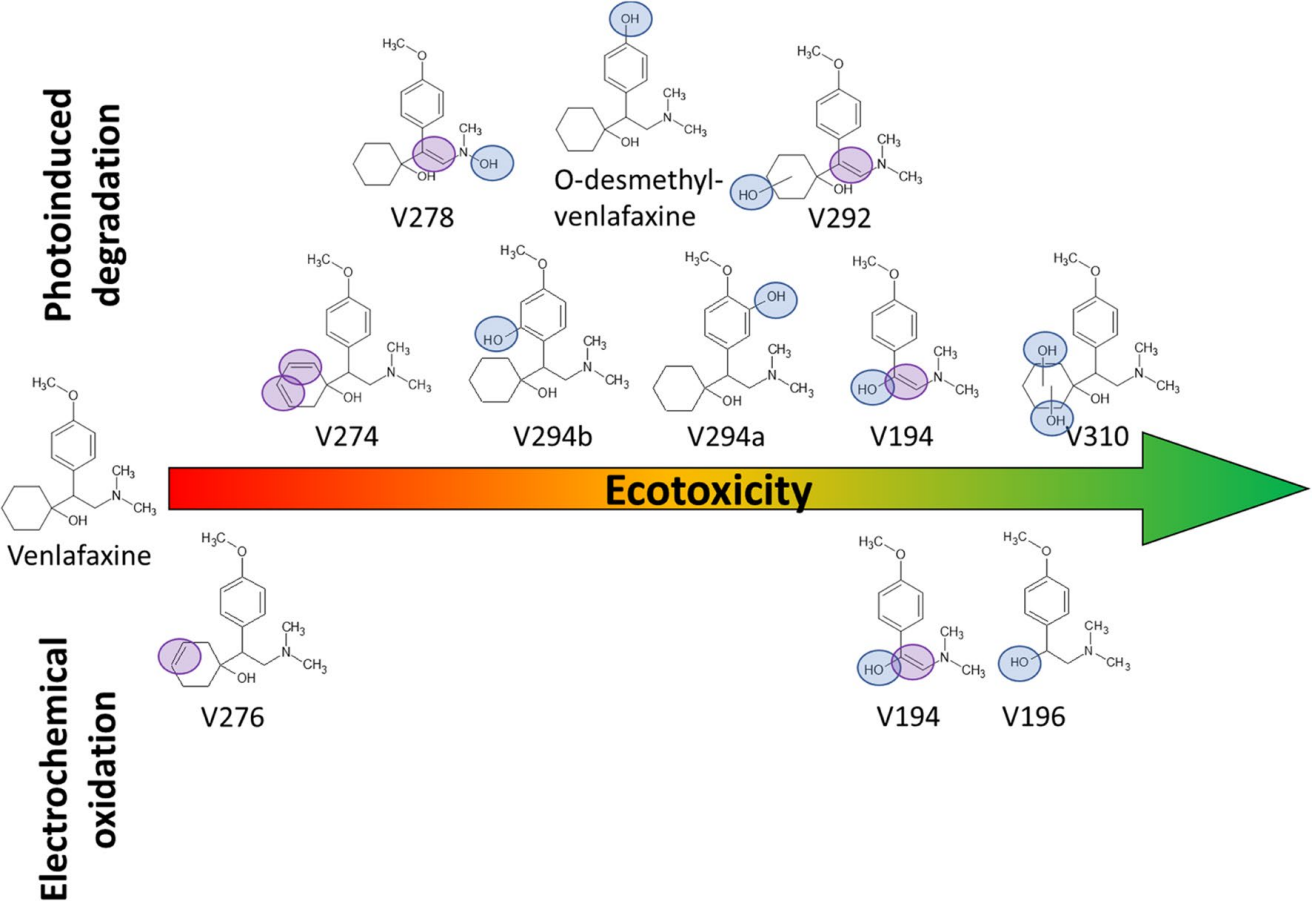
Ecotoxicity-ranking of venlafaxine and its degradation products

To compare the potential ecotoxicological hazard arising from the application of both AOPs, *in silico* QSAR analysis based on the identified structures was performed. Among the different transformation products, the single identical product was Ven194. All ecotoxicological values predicted using ECOSAR are listed in Table S3 of the supplemental information. When no unequivocal identification could be achieved, the hydroxyl group was assumed at different positions. This was taken into account in the QSAR analysis. Details are described in supplemental information. Based on the predicted ecotoxicity values, structures were ranked from higher to lower values as illustrated in Fig. 6.

Except for n-desmethylvenlafaxine, the ECOSAR class Aliphatic Amines (1.0) was applied. With an increasing number of hydroxyl group substituents, the ecotoxicity of the transformation products decreased. A similar finding has been reported for imidacloprid in previous studies (Voigt et al. 2022b). As a consequence, the indirect mechanism, which leads to hydroxyl radical addition or substitution, may be considered the preferential mechanism in terms of ecotoxicity if the conditions might be adjusted in ecological or technical installations. An encouraging finding with respect to environmental hazard was that all products resulting from photoirradiation and electrochemical treatment were predicted less toxic than venlafaxine itself. The most ecotoxic transformation product was V276 resulting from electrochemical generation, followed by V274 and V278 from photoirradiation.

In terms of ecotoxicity, degradation efficiency, and treatment duration, photoirradiation seems advantageous to electrochemical oxidation. Extrapolating from NOM to real wastewater or effluents, electrochemistry might prove more robust. For evaluation of technical scale application however, economic considerations would need to be taken into account as well (Balakrishnan et al. 2021). A reliable forecast cannot be achieved at this stage.

## Conclusion

The photoinduced and electrochemical oxidative degradation of venlafaxine was investigated under varied conditions. Kinetic studies of the initial venlafaxine and the transformation products revealed the efficiency of both AOPs and the effect of the matrix and adjuvants. The structural elucidation of the occurring transformation products was achieved through higher-order mass spectrometry and allowed to gain insight into the degradation mechanisms. While photoinduced degradation proceeded predominantly via the indirect hydroxyl radical–induced pathway, electrochemical oxidation occurred via the direct anodic and hydroxyl radical–mediated mechanism. QSAR analysis predicted that none of the emerging transformation products posed more ecotoxicological hazard than venlafaxine. As a general tendency, hydroxyl groups as substituents lowered the ecotoxicological potential. In total, UV irradiation proved a more efficient means for elimination from the aquatic environment than electrochemical oxidation due to faster degradation. Yet, irradiation was more susceptible to matrix effects from humic acid as a natural organic matter representative indicating potential challenges for projected applications as a fourth purification stage. These challenges as well as those from pH variations in WWTPs may apply equally to photoinduced catalyst-enhanced AOPs. On a larger scale required for an extended purification, both electrochemical and photoirradiation AOPs may prove demanding.

### Supplementary information

ESM 1

## Data Availability

Data can be obtained upon request from the corresponding author.
