# Supplementary material for: Comparison of photoinduced and electrochemically induced degradation of venlafaxine
Source: Environ Sci Pollut Res Int. 2024 Jan 22;31(9):13442–54. doi: 10.1007/s11356-024-32018-5 (PMC10881652; doi:10.1007/s11356-024-32018-5)
Supplement: Supplementary file 1 — ESM 1 [file 11356_2024_32018_MOESM1_ESM.docx]

**Photoinduced and electrochemically induced degradation of venlafaxine**

Melanie Voigt, Jean-Michel Dluziak, Nils Wellen, Victoria Langerbein and Martin Jaeger*

*corresponding author:

Niederrhein University of Applied Sciences, Department of Chemistry and ILOC

Frankenring 20, D-47798 Krefeld, Germany

E-mail: martin.jaeger@hs-niederrhein.de, phone: +49-2151-822-4188

**-Supplemental information -**

## Kinetics of photoinduced degradation and electrochemical oxidation

Concentration-time (*c-t*) diagrams were obtained as m/z-signal areas of initial compounds from the sample series described above. Signal intensities were normalized to the initial concentration of the pesticide. The recorded *c-t* curves were fitted using the curve fitting toolbox within the software MatLab2018a (MathWorks, Natick, MA, USA). All *c-t* curves of imazalil, penconazole and tebuconazole were computed assuming the first-order kinetic model of equation 1.

$A\underset{\to}{k_{1}}B+.. .$ (1)

Venlafaxine reacts to an unknown number of products B, C, D etc. The usual mathematical treatment leads to equation 2 with actual concentration *c*_A_ of the compound during degradation depending on time *t* and the initial concentration *c*_A0_ (Mauser 1974). Equation 2 was used for *c-t* curve fitting.

$c_{A}=c_{A_{0}}\cdot e^{-k_{1}t}$ (2)

Both electrochemical oxidation and UV irradiation can result in products described as follow-up or subsequent follow-up products, see equation 3 and 4. The rate constants of steps 1, 2, and 3 are described as *k_1_*, *k_2_*, and *k_3_* and *c*_n_ is the concentrations of the corresponding products.

$c_{A_{1}}=c_{A_{0}}\cdot\frac{k_{1}}{k_{2}-k_{1}}\cdot(e^{{-k}_{1}t}-e^{{-k}_{2}t})$ (3)

$c_{A_{2}}={(c}_{A_{0}}\cdot k_{1}\cdot k_{2})\cdot\left( \frac{e^{{-k}_{1}t}}{\left( k_{2}-k_{1} \right)\left( k_{3}-k_{1} \right)}-\frac{e^{{-k}_{2}t}}{\left( k_{2}-k_{1} \right)\left( k_{3}-k_{2} \right)}+\frac{e^{{-k}_{3}t}}{\left( k_{3}-k_{1} \right)\left( k_{3}-k_{2} \right)} \right)$ (4)

The half-live *t*_1/2_ was calculated according to Equation 5.

$t_{1/2}=\frac{\ln(2)}{k}$ (5)

**Figure S1 Emission spectrum of the UV source (purple) and absorption spectrum of venlafaxine (green) (SI)**

**
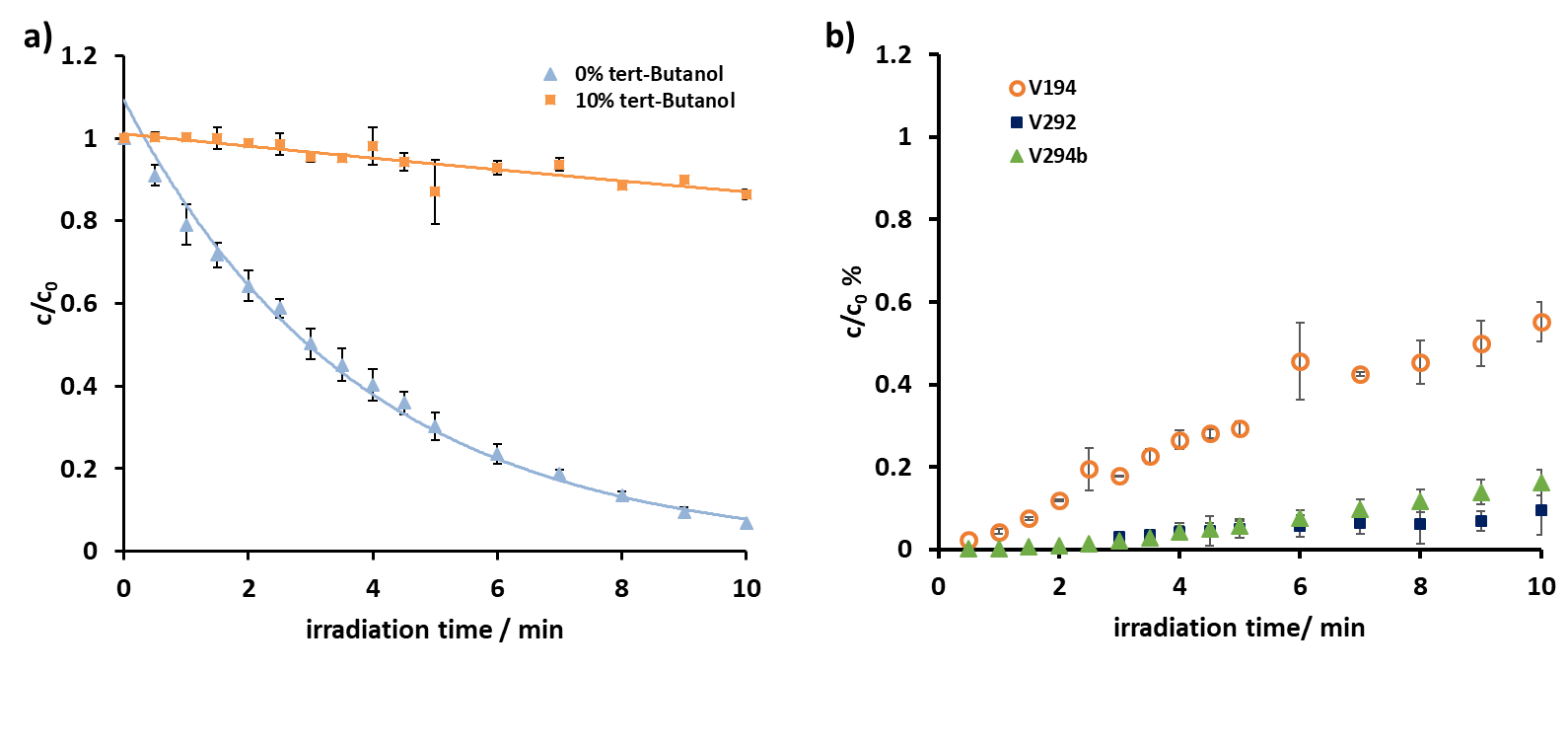
**

**Figure S2 Normalized concentration-time curves of a) venlafaxine in pure water (▲, light blue), in the presence of *tert*-butanol 10 % (■, orange), and b) venlafaxine Ven194 (Ο, orange), Ven292 (■, dark blue), Ven294a (x, red) and Ven294b (▲, green) upon photo-irradiation in the presence of 10% *tert*-butanol; initial concentration of venlafaxine was 20 ± 0.3 mg L^-1^. Error bars from replicate experiments represent standard deviations from replicate experiments.**

**Table S1 Rate-constants k and their half-lives of photoinduced degradation**

| **H_2_O_2_ mg L^-1^** | ***tert*-Butanol /%** | **Humic acid /mg L^-1^** | **k /min^-1^** | **t_1/2_ /min** |
| --- | --- | --- | --- | --- |
| 0 | 0 | 0 | 0.25 | 2.73 |
| 10 | 0 | 0 | 0.50 | 1.38 |
| 30 | 0 | 0 | 0.63 | 1.09 |
| 0 | 10 | 0 | 0.02 | 39.56 |
| 0 | 30 | 0 | 0.02 | 42.55 |
| 0 | 0 | 5 | 0.20 | 3.54 |
| 0 | 0 | 10 | 0.16 | 4.45 |
| 0 | 0 | 30 | 0.12 | 5.79 |

**Simulation of natural organic matter in surface water using humic acid**

**According to DIN EN ISO 7887, the mean value for the absorption coefficient for filtered surface water is 2.40 at 436 nm. Recording absorption spectra of antibiotics in the presence of 5 and 10 mg/L humic acid, 5 mg/L humic acid yielded a comparable optical density, with the exception of erythromycin, and thus represented the best match for surface water, see Table S2.**

**Table S2 Absorption coefficients at 436 nm of solutions containing the investigated antibiotics and humic acid for simulated organic matter in surface water.**

| ****Substance**** | ****5 mg/L humic acid**** | ****10 mg/L humic acid**** |
| --- | --- | --- |
| ****Azithromycin**** | **2.915** | **4.345** |
| ****Erythromycin**** | **3.373** | **4.522** |
| ****Spiramycin**** | **2.639** | **3.594** |
| ****Tylosin**** | **2.446** | **1.331** |

**
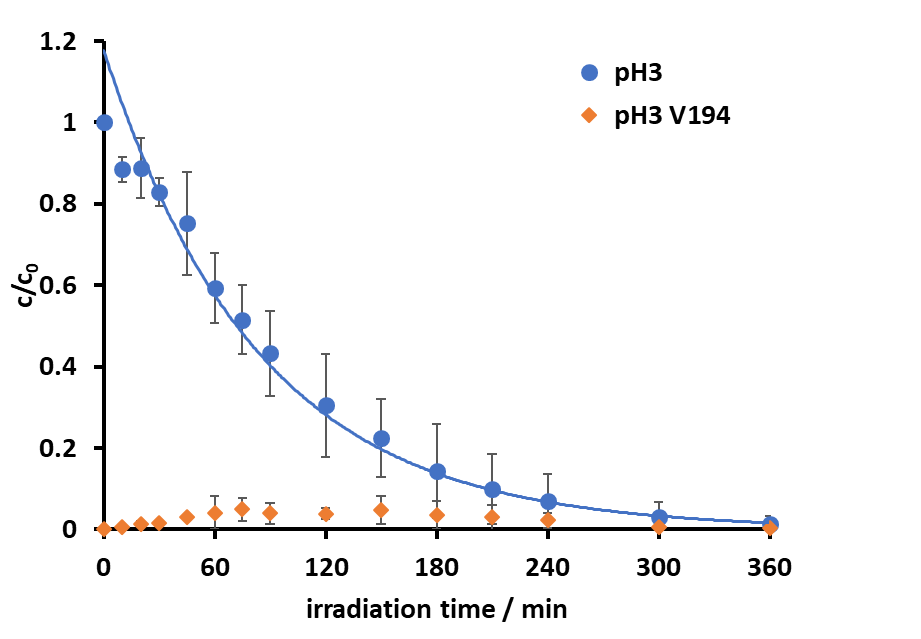
**

**Figure S3. Normalized concentration-time curves of electrochemical oxidation of venlafaxine at a) pH-value 3 (■, light blue) and of V194; initial concentration of venlafaxine was 20 ± 0.3 mg L^-1^. Error bars from replicate experiments represent standard deviations from replicate experiments.**

**QSAR-Analysis**

**For three products, the position of the hydroxyl group in the molecule could not be determined unequivocally. Therefore, QSAR analysis predicted the values for all tentative structures shown in Fig S4.**

**
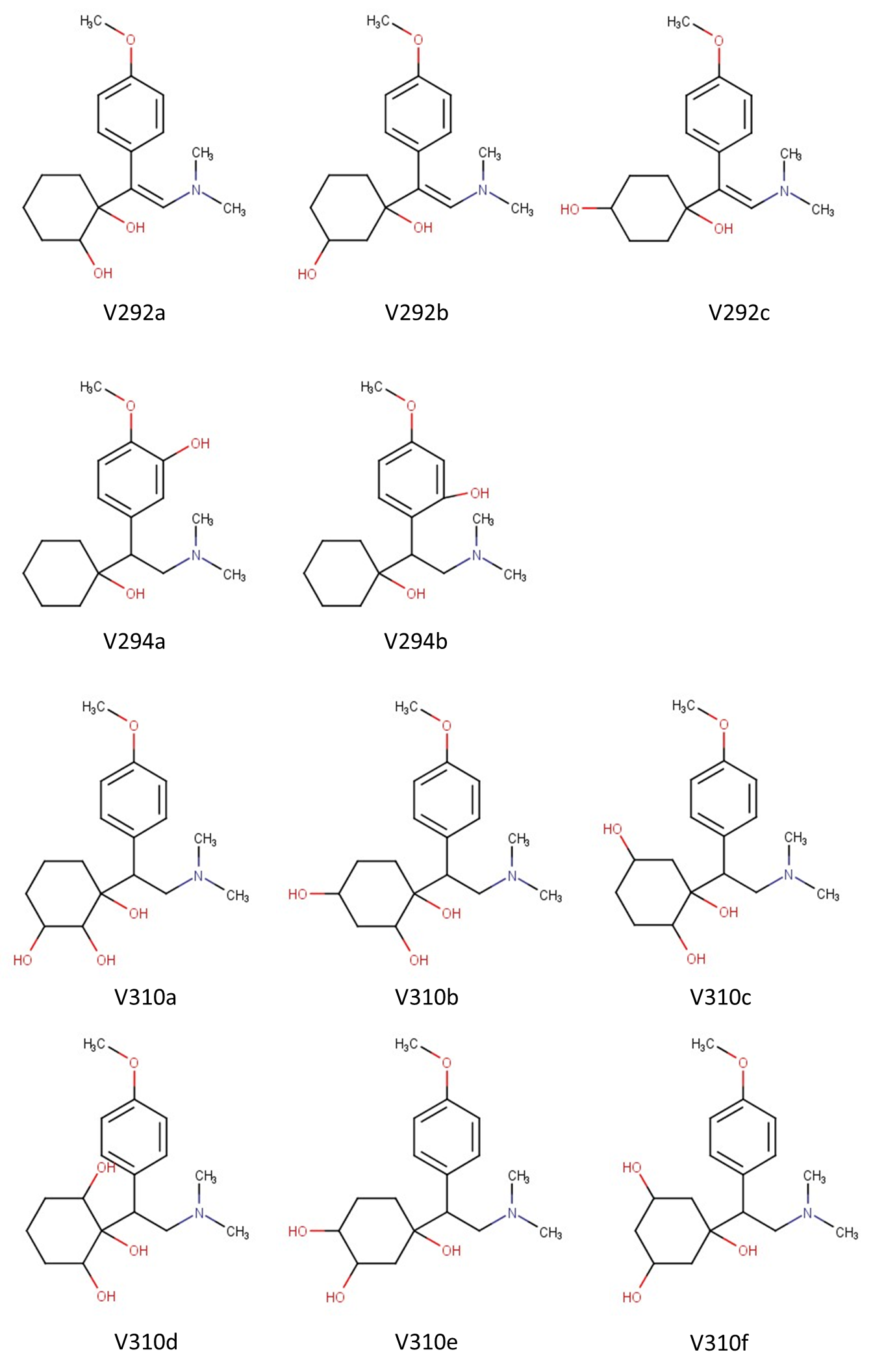
**

**Fig. S4 Structures of identified products with different hydroxyl group positions**

**Table S3 Predicted QSAR-values**

| QSAR name | DAPHNID 48 h LC_50_ Mortality /mg L^-1^ | DAPHNID ChV /mg L^-1^ | FISH 96 h LC_50_ Mortality /mg L^-1^ | FISH ChV /mg L^-1^ | GREEN ALGAE 96 h EC_50_ /mg L^-1^ | GREEN ALGAE ChV /mg L^-1^ |
| --- | --- | --- | --- | --- | --- | --- |
| Venlafaxine | 1.06 | 0.101 | 7.68 | 0.284 | 0.653 | 0.241 |
| V194 | 13.2 | 0.989 | 121 | 9.27 | 13 | 4.04 |
| V196 | 33.4 | 2.33 | 331 | 32.1 | 38.4 | 11.3 |
| O-Desmethyl-Venlafaxine | 2.21 | 0.197 | 17 | 0.768 | 1.54 | 0.544 |
| V274 | 1.91 | 0.173 | 14.5 | 0.625 | 1.3 | 0.462 |
| V276 | 1.42 | 0.131 | 10.5 | 0.418 | 0.913 | 0.331 |
| V278 | 1.99 | 0.18 | 15.2 | 0.659 | 1.36 | 0.483 |
| V292a | 4.45 | 0.378 | 36.1 | 1.9 | 3.43 | 1.17 |
| V292b | 4.45 | 0.378 | 36.1 | 1.9 | 3.43 | 1.17 |
| V292c | 4.45 | 0.378 | 36.1 | 1.9 | 3.43 | 1.17 |
| V294a | 3.16 | 0.276 | 24.9 | 1.2 | 2.3 | 0.8 |
| V294b | 2.2 | 0.198 | 16.8 | 0.737 | 1.51 | 0.536 |
| V310a | 21.7 | 1.62 | 199 | 15.4 | 21.5 | 6.67 |
| V310b | 49.4 | 3.46 | 487 | 46.4 | 56.1 | 16.6 |
| V310c | 49.4 | 3.46 | 487 | 46.4 | 56.1 | 16.6 |
| V310d | 21.7 | 1.62 | 199 | 15.4 | 21.5 | 6.67 |
| V310e | 49.4 | 3.46 | 487 | 46.4 | 56.1 | 16.6 |
| V310f | 49.4 | 3.46 | 487 | 46.4 | 56.1 | 16.6 |

**References**

Mauser H (1974) Formale Kinetik. Experimentelle Methoden der Physik und der Chemie [Formal Kinetics. Experimental Methods of Physics and Chemistry]. Düsseldorf Bertelsmann-Universitätsverlag
